# *Phyllanthus urinaria* Induces Apoptosis in Human Osteosarcoma 143B Cells via Activation of Fas/FasL- and Mitochondria-Mediated Pathways

**DOI:** 10.1155/2012/925824

**Published:** 2012-02-20

**Authors:** Hsin-Yi Wu, Tsu-Kung Lin, Hsiao-Mei Kuo, Ya-Ling Huang, Chia-Wei Liou, Pei-Wen Wang, Jiin-Haur Chuang, Sheng-Teng Huang

**Affiliations:** ^1^Department of Chinese Medicine and Mitochondrial Research Unit, Kaohsiung Chang Gung Memorial Hospital and Chang Gung University College of Medicine, Kaohsiung 833, Taiwan; ^2^Department of Neurology and Mitochondrial Research Unit, Kaohsiung Chang Gung Memorial Hospital and Chang Gung University College of Medicine, Kaohsiung 833, Taiwan; ^3^Mitochondrial Research Unit, Kaohsiung Chang Gung Memorial Hospital and Chang Gung University College of Medicine, Kaohsiung 833, Taiwan; ^4^Department of Internal Medicine and Mitochondrial Research Unit, Kaohsiung Chang Gung Memorial Hospital and Chang Gung University College of Medicine, Kaohsiung 833, Taiwan; ^5^Division of Pediatric Surgery and Mitochondrial Research Unit, Kaohsiung Chang Gung Memorial Hospital and Chang Gung University College of Medicine, Kaohsiung 833, Taiwan

## Abstract

*Phyllanthus urinaria (P. urinaria)*, in this study, was used for the treatment of human osteosarcoma cells, which is one of the tough malignancies with few therapeutic modalities. Herein, we demonstrated that *P. urinaria* inhibited human osteosarcoma 143B cells growth through an apoptotic extrinsic pathway to activate Fas receptor/ligand expression. Both intracellular and mitochondrial reactive oxygen species were increased to lead to alterations of mitochondrial membrane permeability and Bcl-2 family including upregulation of Bid, tBid, and Bax and downregulation of Bcl-2. *P. urinaria* triggered an intrinsic pathway and amplified the caspase cascade to induce apoptosis of 143B cells. However, upregulation of both intracellular and mitochondrial reactive oxygen species and the sequential membrane potential change were less pronounced in the mitochondrial respiratory-defective 143B*ρ* ^0^ cells compared with the 143B cells. This study offers the evidence that mitochondria are essential for the anticancer mechanism induced by *P. urinaria* through both extrinsic and intrinsic pathways.

## 1. Introduction

*Phyllanthus urinaria*, one of the species belonging to the genus *Phyllanthus *(Euphorbiaceae), is used as a traditional folk medicine for the treatment of several diseases including hepatitis B [1], nephrolithiasis [2], and some painful disorders [3]. Recent extensive studies have shown that this title plant exhibits many biological and pharmacological functions *in vitro *and *in vivo*, such as anticancer, cardioprotective, hepatoprotective, antiangiogenic, antioxidant, antisemicarbazide-sensitive amine oxidase, and antihypertensive effects [4–11].

The antitumor effect of *Phyllanthus *species was first verified by its activity against murine B-16 melanoma and P-388 leukemia [8]. Our previous study demonstrated that the aqueous extract of **P. urinaria ** could induce apoptosis in human cancer cells derived from several different origins [12]. Chudapongse et al. reported that **P. urinaria ** could inhibit Hep G2 cell proliferation by acting as an uncoupler and inhibitor of mitochondrial-oxidative phosphorylation [13]. Meanwhile, the water extract of **P. urinaria ** exhibited an anticancer effect in an *in vivo *study not only through apoptosis, but also antiangiogenesis pathways in C57BL/6J mice with implanted Lewis lung carcinoma cells [14]. Tang et al. also proved that *Phyllanthus *species displayed cytotoxic effects on prostate cells and human skin melanoma through modulating the cell cycle and inducing apoptosis [15].

Apoptosis, also called programmed cell death, is a pathway of cell death characterized by several morphological and biochemical events [16]. Cell apoptosis is initiated by extracellular and intracellular signals via two main pathways, the death receptor- and mitochondria-mediated pathways [17]. Mitochondria are commonly involved in the death stimuli through the intrinsic (mitochondrial) pathway of apoptosis, which is the major mechanism of apoptosis in all mammalian cells. This pathway of apoptosis leads to an increase of mitochondrial permeability and the release of proapoptotic molecules (such as cytochrome c, apoptosis-inducing factor and Smac/DIABLO) from the intermembrane space of the mitochondria into the cytosol, thereby activating the caspase-cascade system [18, 19]. Mitochondrial permeability transition (MPT), first characterized by Hunter et al. [20], has been implicated as playing a critical role in the development of apoptotic cell death. Various pathologies can result from oxidative stress-induced apoptotic signaling that is consequent to increases in reactive oxygen species (ROS) and/or decreases in antioxidants, disruption of intracellular redox homeostasis, and irreversible oxidative modifications of lipids, proteins, or DNA [17]. It has been shown that the accumulation of ROS, Ca2+ toxicity, and ischemia damage induce permeability transition pore opening, whereas cyclosporin A (CsA) blocks opening which protects cells from death [21, 22]. It is now well known that the disruption of the apoptotic process through the mitochondria-dependent cell apoptotic pathway is involved in neoplastic transformation and tumor growth [23]. In our previous investigation, the pretreatment with cyclosporine A, an MPT pore inhibitor, reduced *P. urinaria*-induced Lewis lung carcinoma cell apoptosis suggesting that mitochondria are important for the anticancer effect. However, the mechanism of action remains unclear [14].

As we know, osteosarcoma is one of the tough malignancies for which few therapeutic approaches are effective. Therefore, we aimed to determine whether **P. urinaria ** would lead to osteosarcoma cell apoptosis, and approach the relevant molecular mechanisms. We used human osteosarcoma 143B and mitochondrial DNA-deleted 143B*ρ* ^0^ cells to provide evidence that reactive oxidative respiration, mitochondrial membrane potential, and the sequential induction of caspase cascade were required for the antitumor effect of *P. urinaria*.

## 2. Materials and Methods

### 2.1. Preparation of **P. urinaria** Extract

 The **P. urinaria ** used in this study was identified by Dr. Rong-Chi Yang, the chief of the Chinese Herbal Pharmacy in Chang Gung Memorial Hospital, based on the definition described in Flora of Taiwan [24]. The voucher specimen of **P. urinaria ** was deposited and numbered “173156” in the herbarium of National Taiwan University. The extract was prepared according to the quality standards following the GMP (good manufacturing practice) guidelines in Taiwan. The whole plant was minced and mixed with 100°C hot water in the proportion of 1 : 20 (w/v) for 4 hours and repeated for another 4 hours after adding the same amount of water again. The resulting crude extract was filtered and lyophilized down to dry powder. On average, the yield was 26.4% (w/w) from the whole **P. urinaria ** plant. The **P. urinaria ** extract used in the experiments was prepared by dissolving the dry powder extracted from 100 mg of the original whole plant in 1 mL sterile water, filtered and used as a 100 mg/mL stock.

### 2.2. Mass Spectrometry (MS)

High-performance liquid chromatography was performed using a Shimadzu SIL-20A LITE system (Shimadzu Corporation, Columbia, MD, USA). A Shimadzu SPD-10AV UV-Vis Detector was used at *λ* = 270 nm. The chromatographic separation was carried out on an Agilent Zorbax Eclipse XD8-C18 column (4.6 × 150 mm i.d.; 3.5 *μ*m particle size) eluted with mixtures of 0.01% trifluoroacetic acid (aq) (A) and acetonitrile (B). The linear gradient program was set from 95 : 5 (A : B, v/v) to 80 : 20 in 22 min, then the concentration of acetonitrile was increased from 20% to 30% in the following 28 minutes. The flow rate was 0.6 mL/min. Each sample injecting volume was 30 *μ*L. Mass spectra were acquired using an LTQ XL Mass Spectrometer (Thermo Electron Corp, San Jose, CA, USA) equipped with an electrospray ionization source operated in the negative mode using the following conditions: spray voltage (4.5 kV), heated capillary temperature (245°C); capillary voltage 47 V, and tube lens offset (5.25 V). Nitrogen was used as the sheath and auxiliary gas at 35 and 10 units, respectively. MSn experiments were carried out using helium as the collision gas. Data acquisition and analysis were accomplished with Xcalibur software version 2.0 (Thermo Electron Corporation). Gallic acid and ellagic acid (Sigma-Aldrich Corporation, WI, USA) were used as reference compounds and dissolved separately in methanol (MeOH).

### 2.3. Cell Culture and Generation of 143B*ρ* ^0^ Cells

 Human 143B osteosarcoma (purchased from Food Industry Research and Development Institute, Taiwan) were grown in Dulbecco's modified Eagle's medium (DMEM) supplemented with 10% heat-inactivated fetal bovine serum at 37°C in 5% CO2. FBS was thawed at 37°C and then heated to 56°C for 30 min for heat inactivation. Production and culturing of 143B*ρ* ^0^ cells require growth medium containing 1 mM pyruvate and 50 *μ*g/mL uridine (143B*ρ* ^0^ medium) to support growth [25]. The 143B*ρ* ^0^ cells were produced by culturing 143B osteosarcoma cells in the presence of ethidium bromide (EtBr, 50 ng/mL) for 8 weeks. Cells were subcultured every 2-3 days and kept in 50%–70% of confluence. The mtDNA-encoded subunit 2 of cytochrome c oxidase is undetectable for 143B*ρ* ^0^ cells using Western blotting. To examine the effect of **P. urinaria ** extract, cells at 70% confluence were treated with 0~3 mg/mL of **P. urinaria ** extract for 24 and 48 hours.

### 2.4. MTT Assay

 Human osteosarcoma 143B and 143B*ρ* ^0^ cells with or without **P. urinaria ** treatment were washed once with PBS, followed by the addition of 1 mL DMEM containing 0.05 mg/mL 3-(4,5-dimethylthiazol-2-yl)-2 and 5-diphenyltetrazolium bromide (MTT; Sigma). After incubation at 37°C for 1 hour, the media were removed and the formazan crystals in the cells were dissolved in 1 mL DMSO for OD (optical density) reading at 570 nm using a spectrophotometer.

### 2.5. Terminal Deoxynucleotidyl Transferase-Mediated dUTP Nick End-Labeling (TUNEL) Staining

 The TUNEL assay was used to detect DNA fragmentation. 143B and 143B*ρ* ^0^ cells plated on slides with or without **P. urinaria ** treatment for 24 and 48 hours were fixed with 4% methanol-free formaldehyde (pH 7.4) for 5 minutes at 4°C and washed with PBS. TUNEL analysis was performed using an in situ Cell Death Detection Kit Fluorescein (Roche Molecular Biochemicals; Indianapolis, IN) according to the manufacturer's protocol. TUNEL-positive cells were visualized by immunofluorescent microscopy. TUNEL-positive cells containing fluorescence were identified by colocalization with DAPI and by morphology. The slides were viewed under a fluorescence microscope with green fluorescence set at 520 nm. The cells stained green indicated apoptotic cells.

### 2.6. Annexin V-FITC/Propidium Iodide Staining

143B and 143B*ρ* ^0^ cells with or without **P. urinaria ** treatment were harvested and washed twice with cold PBS, then resuspended in 1X binding buffer at a concentration of 6 × 105 cells/mL. 100 *μ*L of the solution (6 × 104 cells) was then transferred to a 5 mL culture tube. All samples were processed for annexin V labeling according to the manufacturer's instructions. Briefly, cells were resuspended in 100 *μ*L of 1x annexin V binding buffer (BD Biosciences) and were fluorescently labeled for the simultaneous detection of apoptotic and necrotic cells by adding 5 *μ*L of annexin V-FITC (BD Biosciences) and 5 *μ*L of 1 mg/mL propidium iodide (PI) to each sample. Samples were gently mixed and incubated at room temperature in the dark for 30 min. At the end of the incubation, 400 *μ*g of 1X binding buffer was added to each sample and the sample was analyzed using a flow cytometer (Becton Dickinson) equipped with CellQuest software.

### 2.7. Detection of ROS

 Intracellular ROS were evaluated by determining the level of hydrogen peroxide (H_2_O_2_) using a 6-carboxy-2, 7-dichlorodihydrofluorescein diacetate (DCFDA) (Sigma) fluorescent probe. In the presence of H_2_O_2_, DCFDA is converted into 2, 7-dichlorodifluorescein (DCF), which can be detected by flow cytometry. Briefly, 6 × 105 cells were plated in six-well plates and allowed to attach for 16–18 hours. After being treated with **P. urinaria ** at the concentration of 0–3 mg/mL for 24 hours or 48 hours, the cells were incubated with 5 *μ*M DCFDA for an additional 30 min, followed by washing and resuspending in PBS. The fluorescence was detected using a BD Biosciences FACScan system.

### 2.8. Mitochondrial ROS Determination

*P. urinaria*-induced mitochondrial superoxide (O_2_^−^) production was quantified by MitoSOX Red, a redox-sensitive dye composed of hydroethidine linked by a hexyl carbon chain to a triphenylphosphonium group to target the mitochondrial matrix because of the negative membrane potential across the inner mitochondrial membrane. 143B and 143B*ρ* ^0^ cells with or without **P. urinaria ** treatment for 24 and 48 hours were washed once with PBS. The cells were then loaded with MitoSOX Red (10 *μ*M) at 37°C for 10 minutes, washed, and used for imaging using immunofluorescence microscopy.

### 2.9. Measurement of Mitochondrial Membrane Potential (ΔΨm)

6 × 10^5^ cells were plated in six-well plates and allowed to attach for 16–18 hours. After being treated with **P. urinaria ** at the concentration of 0–3 mg/mL for 24 hours or 48 hours, the cells were harvested by treatment with trypsin, washed in phosphate-buffered saline (PBS), and resuspended in 200 ng/mL of Rhodamine 123 (Invitrogen). After incubation for 30 min at 37°C, the cells were washed three times and resuspended in 500 *μ*L of PBS. Cytofluorimetric analysis was performed using a fluorescence-activated cell scanner machine (BD Biosciences FACScan system).

### 2.10. Immunofluorescence and Flow Cytometry Analysis

 143B and 143B*ρ* ^0^ cells with or without **P. urinaria ** treatment for 24 and 48 hours were permeabilized using buffer containing 1.5% normal goat serum and 0.2% Triton X-100 in PBS, incubated with Fas and FasL antibodies (1 : 100) for 1 hour, then incubated with Alexa 546 or 488-conjugated secondary antibodies for 30 minutes at room temperature. DAPI was used to stain the DNA/nuclei before mounting in antifade media, and the slides were visualized under a fluorescence microscope (1000X). The surface FasL expressions in the 143B and 143B*ρ* ^0^ cells were determined by flow cytometry analysis. Cells with or without **P. urinaria ** treatment for 24 and 48 hours were trypsinized and incubated with FasL antibody (1 : 100) in Flow Cytometry Staining Buffer (eBioscience; San Diego, CA, USA) at 37°C for 1 hour. After washing with PBS twice, the cells were incubated with Alexa 488-conjugated secondary antibodies (1 : 200) at 37°C for 1 hour, washed with PBS twice, and resuspended in PBS for analysis in a flow cytometer (BD Biosciences, San Jose, CA, USA).

### 2.11. Western Blot Analysis

 The cell lysates from 143B and 143B*ρ* ^0^ cells with or without treatment of **P. urinaria ** were obtained, and protein concentrations were determined by the Bradford method (Bio-Rad, CA, USA). Samples with equal amount of proteins were subjected to 15% sodium dodecyl sulfate polyacrylamide gel electrophoresis (SDS-PAGE) and transferred onto a polyvinylidene difluoride (PVDF) (Millipore, Bedford, MA, USA) membrane. The membrane was incubated at room temperature in blocking solution (10% nonfat milk) for 1 hour, followed by incubation for 2 hours in blocking solution containing an appropriate dilution (1 : 1000) of primary antibody, for example, anti-cleaved caspase-3, anti-cleaved caspase-8, anti-cleaved caspase-9, anti-Bax, anti-Bcl-2, and anti-Bid antibodies (Cell Signaling Technology). After washing, the membrane was incubated in PBS containing goat anti-rabbit IgG conjugated with horseradish peroxidase (Sigma, St. Louis, MO, USA) for 1 hour. The membrane was washed and the positive signals were developed with an Enhanced Chemiluminescence (ECL) system (Amersham Pharmacia Biotech). Membranes were exposed to Fuji medical X-ray film (Fuji Ltd., Tokyo, Japan) for 30 minutes. The *β*-actin expression was used as the internal control.

### 2.12. Statistical Analysis

 All statistical analyses are performed using SigmaStat statistical software (version 2.0, Jandel Scientific, CA, USA). Results are represented as means ± standard deviation (SD). ANOVA was carried out when multiple comparisons were evaluated. Values were considered to be significant at a *P* value less than 0.05. All experiments were repeated at least three times independently.

## 3. Results

### 3.1. Chemical Chromatography of the Aqueous Extract of *P. urinaria*

Analysis of the water extracts of **P. urinaria ** by high performance liquid chromatography (HPLC) led to the identification of twelve compounds as shown in Table 1 [26]. The mass structures of these compounds were tentatively assigned based on mass data mining from existing literature. Compound 1 (*Rt *at 5.8 min) was determined to be 170 by liquid chromatography electrospray ionization tandem mass spectrometry (LC/(-)ESI-MS), yielding [M-H]-at *m/z *169. The major fragment was shown at *m/z *151 (M-H-H2O) and 125 (M-COOH), consistent with the structure of gallic acid [27]. Compound 3 (*Rt *at 18.0 min) and compound 9 (*Rt *at 23.3 min) were determined to be 292 and 248, yielding [M-H]-at *m/z *291 and 247, respectively. The major fragment of compound 3 was shown at *m/z *247 (M-COOH), and the minor fragment ions were the same as compound 9. Thus, the determined mass of compound 3 was 44 higher than compound 9, suggesting a carboxylic acid derivative of compound 9. By searching the bioactive components previously found in *Phyllanthus *species [3, 27], compound 3 and compound 9 were assigned as brevifolin carboxylic acid and brevifolin, respectively. LC/(-)ESI-MS analysis of compound 4 (*Rt *at 19.0 min) produced the same [M-H]-at m/z 291 as compound 3. (-)CAD-MS/MS (collision activated decomposition mass spectrum) analysis of compound 4 yielded intense product ions at *m*/*z *247 (M-COOH), 203 (M-2COOH). Compared with the mass and mass fragments of compounds found in other *Phyllanthus *species, the structure of compound 4 was assigned as phyllanthusiin E [28]. LC/(-)ESI-MS analysis of compound 5 (*Rt *at 19.5 min), compound 6 (*Rt *at 20.5 min), compound 7 (*Rt *at 21.2 min), compound 8 (*Rt *at 22.8 min), compound 10 (*Rt *at 24.2 min), and compound 11 (*Rt *at 24.6 min) yielded [M-H]- at *m/z *633, 951, 953, 925, 969, and 924, respectively. (-)CAD-MS/MS analysis of all these components yielded intense product ions at *m/z *633 and 301, resulting from the loss of the hexahydroxydiphenoyl and/or galloyl moiety fragments [29]. These ions are significant fragments of ellagitannins. After comparing the mass and other minor fragment ions with tannins found in other *Phyllanthus *species, the structures of these compounds were assigned as corilagin [27, 29], geraniin [30], chebulagic acid [31], phyllanthusiin C [32], phyllanthusiin B [32], and phyllanthusiin U [27], respectively.

Compound 2 (*Rt *at ca. 14.0 min) produced the same [M-H]-at m/z 633 as compound 5. The fragment ions at *m/z *453 (M-H-Gal) and 301 (M-H-Gal-glc), corresponding to compound 5, were consistent with the structure of isostrictinin [33]. Compound 12 (*Rt *at 28.7 min) yielded an [M-H]-at *m/z *301, consistent with a mass of 302. The same fragment pattern was shown by comparing (-)CAD-MS/MS analysis of compound 12 with the product ion spectral analysis of the fragment ion at *m/z *301 (MS3) in compound 5 (corilagin). This suggested an ellagic acid moiety, and compared with the fragments in the literature [26], compound 12 was assigned as ellagic acid. The identities of compound 1 and compound 12 were further confirmed by chemical markers (mass and HPLC retention times) to be gallic and ellagic acid, respectively.

### 3.2. Cell Viability of Osteosarcoma 143B and 143B*ρ* ^0^ Cells Treated with *P. urinaria*

**Figure 1:**
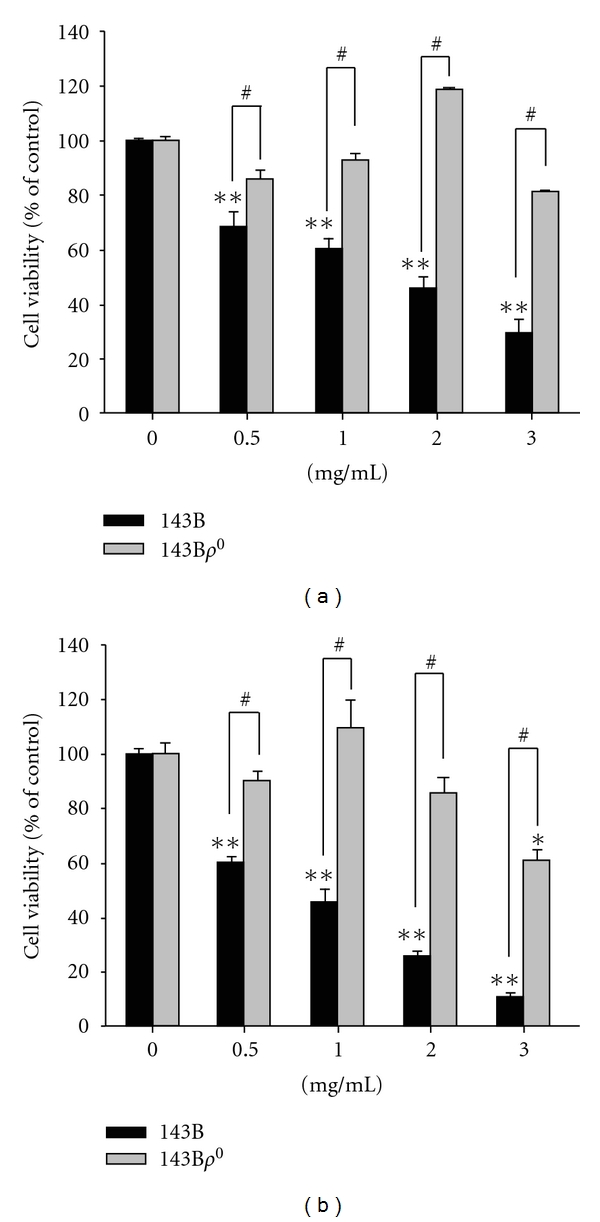
Cell viability of **P. urinaria ** extract on 143B and 143B*ρ* ^0^ cells. Cells were treated with different concentrations for (a) 24 h and (b) 48 h. The cell viability was determined by standard MTT assay. The bar value is the mean ± SD of three independent experiments in duplicate. Asterisks mean statistical significance in comparison with the vehicle control (*P < 0.05, **P < 0.01). A pound sign represents a significant difference between 143B and 143B*ρ* ^0^ cells (^#^ *P* < 0.05).

The cell viability of 143B cells was significantly inhibited by **P. urinaria ** treatment in a dose-dependent manner. **P. urinaria ** extract at concentrations of 2 mg/mL for 24 hours and 1 mg/mL for 48 hours caused an approximate 50% decrease (Figures 1(a) and 1(b)), respectively, in 143B cells compared to vehicle controls. Nearly complete loss of 143B and 143B*ρ* ^0^ cells was observed with a higher dose or longer exposure (>72 hours) to **P. urinaria ** treatment. 143B*ρ* ^0^ cells, which lack mitochondrial DNA-derived from the human osteosarcoma cell line 143B, were introduced to compare the cytotoxic effect of *P. urinaria*. 143B*ρ* ^0^ cells were significantly more resistant to the cell-killing effect of **P. urinaria ** extract following 24 hours and 48 hours treatment, compared with the parental cell line (143B) under identical culture conditions (Figures 1(a) and 1(b)). This indicated that a functional respiratory chain and active oxidative respiration were required for the cytotoxic effect induced by *P. urinaria*.

### 3.3. Induction of Apoptosis by *P. urinaria*

**Figure 2:**
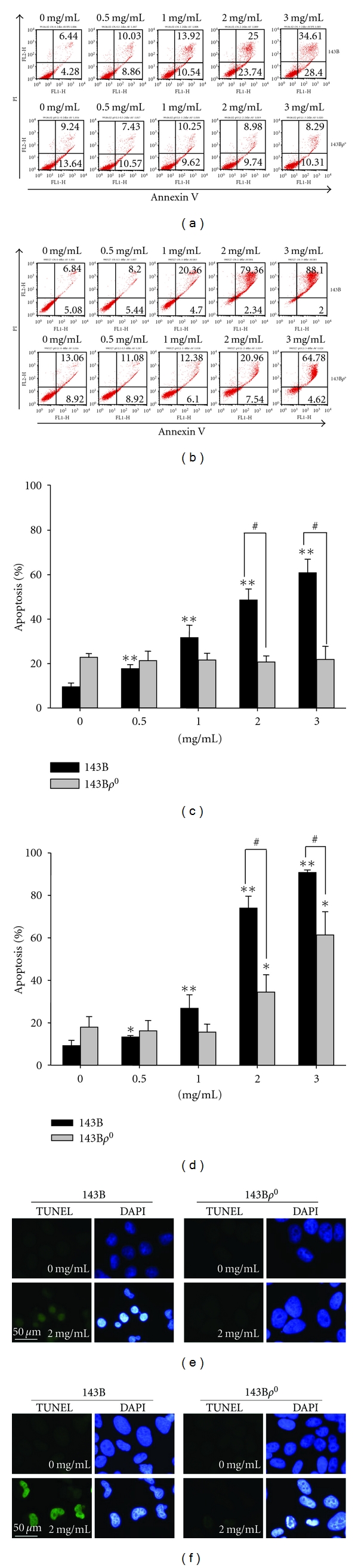
Apoptosis of 143B and 143B*ρ* ^0^ cells of **P. urinaria ** detected by flow cytometry with annexin V and propidium iodide (PI) and TUNEL staining. 143B and 143B*ρ* ^0^ cells treated with **P. urinaria ** for 24 h (a) and 48 h (b) are shown with representative dot plots from FITC-conjugated annexin V and PI staining. Early apoptosis was recognized in the presence of staining for annexin V and absence of staining for PI. Late apoptosis was recognized in the presence of staining for both annexin V and PI. The percentage of apoptosis from flow cytometric analysis for 24 h (c) and 48 h (d) was assessed. Values are mean ± SD of three independent experiments in duplicate. Asterisks represent statistical significance from the vehicle control (*P < 0.05, **P < 0.01). A pound sign represents statistical significance between 143B and 143 B*ρ* ^0^ cells (^#^ *P* < 0.05). Immunofluorescence showing apoptotic 143B and 143B*ρ* ^0^ cells marked by TUNEL assay in the absence and presence of 2 mg/mL **P. urinaria ** treatment for 24 h (e) and 48 h (f) are indicated. DAPI staining was used to detect all nuclei and visualized under a fluorescence microscope (1000x). DNA damage of 143B cells in comparison with 143 B*ρ* ^0^ cells was markedly increased with the treatment of **P. urinaria ** (^#^ *P* < 0.05).

It is well known that a cytotoxic effect is associated with intrinsic and extrinsic stimulations to result in apoptosis. In order to confirm apoptosis induced by *P. urinaria*, we next determined whether 143B and 143B*ρ* ^0^ cells displayed a differential sensitivity to *P. urinaria*-induced apoptosis through annexin V/PI staining and TUNEL assay. As shown in Figures 2(a) and 2(b), the apoptotic cell count of 143B cells was significantly increased by **P. urinaria ** treatment after incubation for 24 and 48 hours by staining with annexin V, which binds to phosphatidylserine with high affinity. We also compared the cytotoxic effects of **P. urinaria ** on 143B*ρ* ^0^ cells. The results showed that **P. urinaria ** exhibited no cytotoxic effect on 143B*ρ* ^0^ cells with 3 mg/mL **P. urinaria ** treatment for 24 hours incubation (Figure 2(a)). However, a concentration higher than 2 mg/mL of **P. urinaria ** with 48 hours treatment did inhibit 143B*ρ* ^0^ cell growth, but less than for 143B cells (Figure 2(b)). This suggested that parental 143B cells were more sensitive to *P. urinaria*-induced apoptosis than 143B*ρ* ^0^ cells (Figures 2(c) and 2(d)). DNA fragmentation, which is the hallmark of apoptosis, by TUNEL and DAPI staining used to detect all nuclei was introduced to further analyze 143B and 143B*ρ* ^0^ cells with **P. urinaria ** treatment. The results demonstrated a significant increase of apoptosis in 143B cells with a higher concentration and longer exposure to **P. urinaria ** treatment than in 143B*ρ* ^0^ cells (Figures 2(e) and 2(f)). Taken together, our results indicated that the amount of apoptosis in 143B cells was markedly increased in response to **P. urinaria ** treatment compared to 143B*ρ* ^0^ cells.

### 3.4. Change of Intracellular ROS Levels in 143B and 143B*ρ* ^0^ Cells

**Figure 3:**
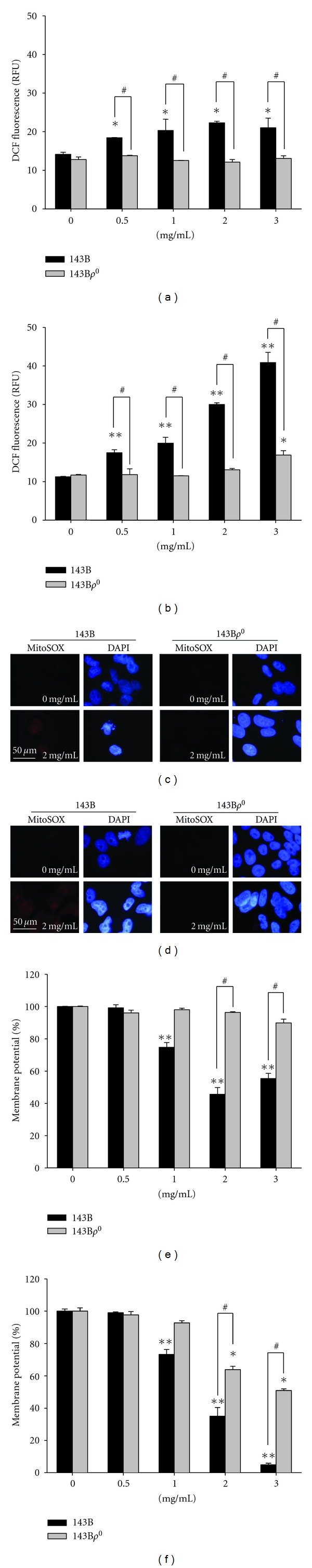
Effects of 143B and 143B*ρ* ^0^ cells with *P. urinaria*-induced intracellular and mitochondrial ROS generation and mitochondrial membrane potential. The intracellular ROS with DCFDA staining by FACS analysis in 143B and 143B*ρ* ^0^ cells for 24 (a) and 48 (b) hours. The values are displayed as the mean ± SD of the mean fluorescence intensity of DCF. 143B and 143B*ρ* ^0^ cells were preincubated with MitoSOX Red and DAPI in the absence or presence of **P. urinaria ** 2 mg/mL treatment for 24 (c) and 48 (d) hours. Representative digital images of MitoSOX and DAPI fluorescence were obtained using a fluorescence microscope (1000x). The mitochondrial membrane potential (ΔΨm) stained with Rhodamine 123 by FACS analysis in 143B and 143B*ρ* ^0^ cells for 24 (e) and 48 (f) hours. The values are displayed as the mean ± SD of percent control on intracellular ROS and mitochondrial membrane potential. Three independent experiments in duplicate were performed. Asterisks represent statistical significance from the vehicle control (*P < 0.05, **P < 0.01). A pound sign represents statistical significance between 143B and 143B*ρ* ^0^ cells (^#^ *P* < 0.05).

Recently, **P. urinaria ** has been reported to inhibit HepG2 cells by acting as an inhibitor of oxidative phosphorylation and a weak mitochondrial uncoupler [13]. To address whether **P. urinaria ** enhances ROS generation in 143B and 143B*ρ* ^0^ cells, the intracellular H_2_O_2_ concentration was detected by DCFDA-based flow cytometric analysis. As shown in Figures 3(a) and 3(b), the more significant shift of mean fluorescence intensity (MFI) in 143B cells with **P. urinaria ** treatment demonstrated enhancement of ROS levels in a both dose- and time-dependent manner, but not in 143B*ρ* ^0^ cells. This indicated that the generation of intracellular ROS increased in 143B cells with **P. urinaria ** treatment compared to 143B*ρ* ^0^ cells. As the relevance of the ROS-generating sites in mitochondria may be different from those producing ROS in living cells, we further examined the levels of mitochondrial superoxide with MitoSOX fluorescence. The results demonstrated that **P. urinaria ** triggered superoxide production in mitochondria to inhibit cell proliferation in 143B cells, but to a lesser extent in 143B*ρ* ^0^ cells (Figures 3(c) and 3(d)).

### 3.5. Decreased Mitochondrial Membrane Potential in 143B and 143B*ρ* ^0^ Cells

In our previous study [14], we found that *P. urinaria*-induced apoptosis might be correlated with the loss of mitochondrial transmembrane potential to activate the mitochondria-dependent intrinsic pathway. In this study, **P. urinaria ** resulted in a uniform reduction in mitochondrial membrane potential in parental 143B cells in a time- and dose-dependent manner (Figures 3(e) and 3(f)). Maintenance of mitochondrial potential of 143 B*ρ* ^0^ cells was unaffected by incubation with **P. urinaria ** for 24 hours. However, a decrease of mitochondrial membrane potential of 143B*ρ* ^0^ cells was noted in response to 2 or 3 mg/mL incubation with **P. urinaria ** for 48 hours, but not as prominently in comparison with 143B cells (Figures 3(e) and 3(f)).

### 3.6. Effects of *P. urinaria* on the Protein Expressions of Fas Receptor and Fas Ligand

**Figure 4:**
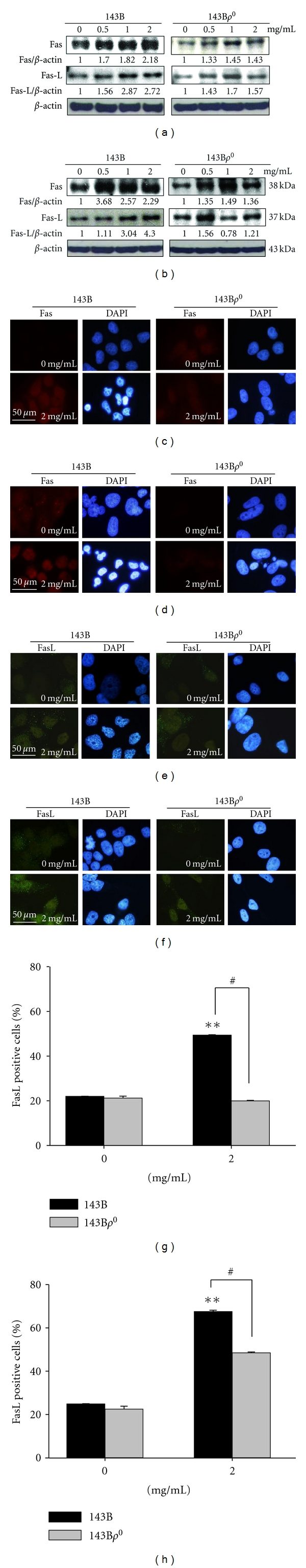
**P. urinaria ** enhanced the Fas and FasL expression in 143B cells, but to a lesser extent in 143B*ρ* ^0^ cells. Cell lysates treated with **P. urinaria ** for 24 hours (a) and 48 hour (b) were subjected to Western blot analysis. Cells were pretreated with **P. urinaria ** for 24 (c, e) and 48 (d, f) hours and incubated with Fas or FasL antibodies one hour before harvest and then stained for Fas or FasL with anti-IgG3 Alexa 546- or 488-conjugated secondary antibodies (red or green). DAPI staining was used to detect all nuclei and visualized under a fluorescence microscope (1000x). Flow cytometry analysis of surface FasL. Quantitation of surface FasL levels in 143 B and 143B*ρ* ^0^ cells treated with **P. urinaria ** for 24 (g) and 48 (h) hours after Alexa 488-conjugated secondary antibodies was determined by flow cytometry analysis. An asterisk represents statistical significance from the vehicle control (**P < 0.01). A pound sign represents statistical significance between 143B and 143B*ρ* ^0^ cells (^#^ *P* < 0.05).

The critical elements of the Fas pathway that link receptor/ligand interaction and downstream activation of caspases including caspase-3 were identified in our previous report [34]. To further elucidate the molecular mechanism underlying the *P. urinaria*-induced apoptosis in 143B and 143B*ρ* ^0^ cells, we examined the protein expressions of Fas receptor/ligand by Western blot, immunofluorescence, and flow cytometry analysis. The protein expressions of both Fas receptor and ligand were induced by the treatment of **P. urinaria ** in 143B cells. However, Fas receptor and ligand were triggered less prominently by the treatment of **P. urinaria ** in 143B*ρ* ^0^ cells (Figures 4(a) and 4(b)), whereas both Fas receptor and ligand expressions were increased at the surface of 143B cells, but to the lesser extent in 143B*ρ* ^0^ cells (Figures 4(c), 4(d), 4(e), 4(f)). Flow cytometry analysis of the Fas ligand in 143B and 143*ρ* ^0^ cells exposed to 2 mg/mL **P. urinaria ** confirmed the Western blot and immunofluorescence data (Figures 4(g) and 4(h)). These observations indicated that *P. urinaria*-induced death was dependent upon Fas receptor/ligand interactions through an extrinsic pathway.

### 3.7. Effects of *P. urinaria* on the Protein Expressions of Bcl-2 Family and Cysteine-Aspartic Proteases in 143B and 143B*ρ* ^0^ Cells

**Figure 5:**
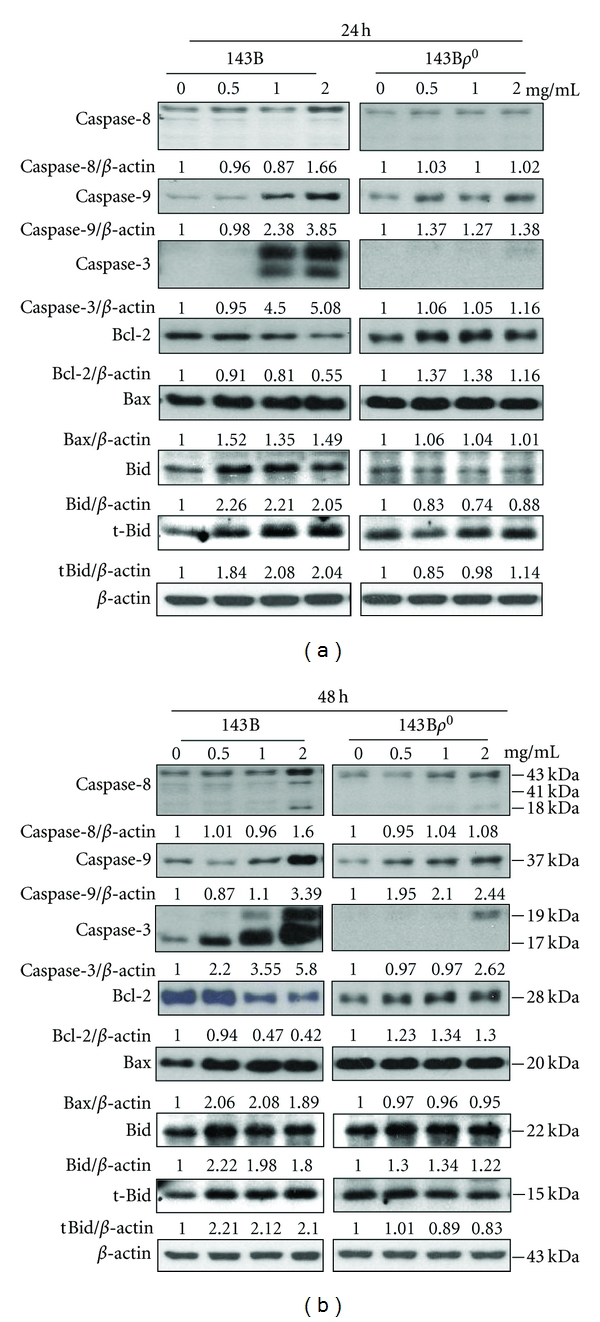
The effects of apoptosis-related protein expression in 143B and 143B*ρ* ^0^ cells. Cells were treated with the indicated concentrations of **P. urinaria ** for 24 hours (a) and 48 hours (b). Cell lysates were subjected to Western blot analysis using antibodies including caspase-3, caspase 8, caspase 9, Bax, Bcl-2, Bid, tBid and *β*-actin antibodies. The Western blot data represent one of the three independent experiments.

**Figure 6:**
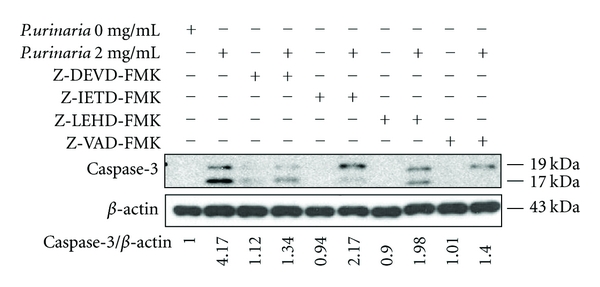
Caspase inhibitors decreased apoptosis inducing by **P. urinaria ** in 143B cells. The representative Western blot analyses demonstrating the effects of the caspase-3, 8, 9 inhibitors including Z-DEVD-FMK, Z-IETD-FMK, Z-LEHD-FMK and the pan-caspase inhibitor Z-VAD-FMK on caspase-3 expression in 143B cells treated without or with 2 mg/mL *P. urinaria*. The Western blot data represent one of the three independent experiments.

To further investigate the molecular mechanism responsible for the *P. urinaria*-induced apoptosis in 143B and 143B*ρ* ^0^ cells, the protein expression of the Bcl-2 family was analyzed by Western blot. Members of the Bcl-2 family, including proapoptotic (Bid, Bax, and Bak) and anti-apoptotic (Bcl-2 and Bcl-xL) proteins, are critical regulators of the intrinsic pathway to modulate the permeabilization of mitochondrial membranes [14]. **P. urinaria ** treatment led to the upregulation of proapoptotic protein expression such as Bax, Bid, while apoptotic proteins including Bcl-2 were downregulated in a time- and dose-dependent manner (Figures 5(a) and 5(b)), which therefore resulted in the relative increase of Bax/Bcl-2 ratio in 143B cells, but not obviously in 143B*ρ* ^0^ cells. Additionally, the induction of apoptosis typically leads to the activation of the caspase cascade. We demonstrated cleaved caspase 3, 8, and 9 activation induced by **P. urinaria ** in 143B cells (Figures 5(a) and 5(b)); however, cleaved caspase 3, 8, and 9 in 143B*ρ* ^0^ cells treated with **P. urinaria ** were less activated in comparison with 143B cells. Apoptosis induced through the Fas receptor/ligand activates caspase-8 and leads to the release of the caspase-8 active fragments. Upon apoptotic stimulation, cytochrome c released from mitochondria associates with the 47 kDa procaspase-9/Apaf 1. Apaf-1-mediated activation of caspase-9 involves intrinsic proteolytic process. Additional cleavage occurs at Asp330 producing 37 kDa subunit that can serve to amplify the apoptotic response. To better correlate caspase activation with induction of apoptotic cell death, we examined caspase-3 expression in 143B cells treated with the caspase-3 inhibitor Z-DEVD-fmk (10 *μ*mol/L), caspase-8 inhibitor Z-IETD-fmk (10 *μ*mol/L), caspase-9 inhibitor Z-LEHD-fmk (10 *μ*mol/L), and pan caspase inhibitor Z-VAD-fmk (10 *μ*mol/L) for 2 hours, followed by with or without 2 mg/mL of **P. urinaria ** for 24 hours. Pretreatment with caspase inhibitors sharply suppressed caspase-3 expression in Western blots (Figure 6).

## 4. Discussion

Numerous naturally occurring botanic extracts are recognized to be antioxidants, cancer preventive agents, or are even used as a cancer therapy drugs such as paclitaxel and vincristine [35]. It is now well established that a disruption of the apoptotic process is involved in neoplastic transformation and tumor growth [23]. In recent years, there has been increasing interest in many compounds from medicinal or dietary plants which possess potential chemopreventive properties. For example, shikonin, honokiol, gallic acid, and ellagic acid have all been shown to inhibit tumor proliferation in diverse cell model [26, 36–38]. However, consistency in composition and biological activity are essential requirements for the safe and effective application of medications. We used high-performance liquid chromatography-mass spectrometry (HPLC/MS) to characterize the twelve compounds in **P. urinaria ** as a fingerprint and to ascertain batch-to-batch quality control (Table 1). **P. urinaria ** has also been reported in many studies to possess antitumor activity [4, 6, 12, 14, 39]. In our previous investigations, **P. urinaria ** inhibited many human cancer cells including HL-60, Molt-3, HT 1080, K-562, HepG2, and NPC-BM1 *in vitro* [13, 14] with morphological changes and DNA fragmentation, but not normal cells including endothelial cells (HUVECs) and liver cells (WRL68) [12]. Based on our former research, gallic acid and ellagic acid were supposed to be the most effective compounds in the **P. urinaria ** to induce the cytotoxic effect of NPC-BM1 cells [37]. In the present study, apoptosis of osteosarcoma 143B cells was also triggered by *P. urinaria*.

In our previous report [34], we found that the gene expressions of both Fas receptor and ligand were increased by the treatment of **P. urinaria ** in HL-60 cells and tightly associated with the event of apoptosis through a ceramide-related pathway. The Fas receptor/ligand signaling system [40] is an important mediator of cell apoptosis by extrinsic stimulation. By the Fas receptor-ligand linkage, Fas activates apoptotic signaling through a cytoplasmic death domain that interacts with signaling adaptors such as Fas-associated protein with death domain (FADD) to play a critical role in the downstream activation of the caspase cascade. Procaspase-8 binds to Fas-bound FADD leading to the activation of caspase-8 to stimulate a caspase cascade and subsequently leads to cell dismantling, DNA degradation, and ultimately cell death. Herein, we demonstrated that **P. urinaria ** triggered apoptosis of 143B cells through Fas receptor/ligand expressions and induced downstream caspase 3, 8, and 9 activation. This indicates that **P. urinaria ** induced apoptosis through both intrinsic and extrinsic mechanisms. Additionally, using the mitochondrial defective 143B*ρ* ^0^ cells, we provided supporting evidence that mitochondrial respiratory function and the intrinsic pathway may be essential for the anticancer effect of *P. urinaria*.

In the present study, we demonstrated increasing protein levels of tBid, Bid, and Bax, and decreasing protein levels of Bcl-2. In the intrinsic pathway, a death signal can also be induced by Fas receptor/ligand on the cell membrane and activate apoptotic signals, which cause procaspase-8 cleavage into caspase-8. Then, caspase-8 activates caspase-3 directly or triggers mitochondrial signal amplification such as the Bcl-2 family [41–43] to disrupt transmembrane potential and open the mitochondrial permeability transition pores responsible for releasing apoptogenic factors, such as cytochrome C, into the cytosol [41]. Following cleavage by caspase-8, the BH3-only protein, Bid, is known to activate Bax [42]. Bid is located in the cytosol and is activated by caspase-8 cleavage upon engagement of cell surface death receptors to stimulate Bax translocation to the mitochondrion regulated by tBid. Then, it undergoes oligomerization to induce the release of cytochrome C to the cytosol to activate caspase-9 and caspase-3. Bcl-2 proteins predominantly localize on the outer mitochondrial membrane, and mediate antiapoptotic effects by stabilizing the mitochondrial membrane, inhibiting permeability transition pore ability and the release of cytochrome c [21]. The balance of proapoptotic Bax and antiapoptotic Bcl-2 is known to be important in determining whether cells die or survive. The Bax/Bcl-2 ratio in a cell acts to regulate its own susceptibility to apoptosis [44]. Therefore, the relative increase of apoptotic Bax/Bcl-2 ratio was correlated well with *P. urinaria*-induced apoptosis in 143B cells.

Mitochondrial membrane potential change can result from oxidative stress-induced apoptotic signaling that is consequent to ROS increases and/or antioxidant decreases, disruption of intracellular redox homeostasis, and irreversible oxidative modifications of lipids, proteins, or DNA [17]. To further clarify the correlation between **P. urinaria ** and mitochondria, we used DCF fluorescence and MitoSOX (mitochondria-targeted superoxide-specific fluorescent probe) Red to determine the intracellular and mitochondrial ROS in this study. Both intracellular and mitochondrial ROS were increased after treatment with *P. urinaria*. This result may provide evidence that an accumulation of reactive oxygen species can induce MPT pore opening to induce apoptosis through a mitochondria-associated (intrinsic) pathway, which is compatible with our previous results where cyclosporin A blocked opening which protected cells from death [14, 21, 22]. In contrast, **P. urinaria ** failed to induce intracellular and mitochondrial ROS and subsequently to decrease membrane potential in 143B*ρ* ^0^ cells, indicating that mitochondrial respiration plays an important role in inducing cell death by *P. urinaria*.

Based on the present results and previous findings, we have summarized the apoptotic signaling pathway by **P. urinaria ** in human osteosarcoma 143B cells. Initially, **P. urinaria ** triggers Fas ligand binding with its receptor through an intracellular adaptor protein (FADD) to activate caspase-8. The activated caspase-8 truncates Bid to activate Bax that binds with the outer membrane of the mitochondrion and is able to open the mitochondrial permeability transition pore in the membrane to release cytochrome C to the cytosol. The balance of proapoptotic Bax and antiapoptotic Bcl-2 was also associated with apoptosis. An increase of the Bax/Bcl-2 ratio acted to regulate its own susceptibility to amplify the mitochondrial apoptotic pathway to release caspase-9 and downstream effecter caspase-3 to induce apoptosis of 143B cells. Furthermore, *P. urinaria*-induced ROS generation and apoptosis were significantly attenuated in mitochondrial function-deficient (143B*ρ* ^0^) cells. This suggests that the ability of **P. urinaria ** to induce 143B apoptosis might be closely related to normal mitochondrial function. Taken together, the mechanism of the anti-tumor effect by **P. urinaria ** was modulated by apoptosis through both extrinsic and intrinsic pathways. Our results also indicate the importance of intact mtDNA for the mechanism of the anti-tumor effect by **P. urinaria ** and might contribute to effective therapeutic strategies for osteosarcoma, which is one of tough malignancies with few effective treatments.

##  Author's Contributions

Hsin-Yi Wu and Tsu-Kung Lin contributed equally to this work.

## Figures and Tables

**Table 1:** Fingerprint of* P. urinaria*.

Compound	Retention time (min)	[M-H] m/z	MS-MS fragmentation
Gallic acid (1)	5.8	169	125, 151
Brevifolin carboxylic acid (3)	18	291	247, 291
Brevifolin (9)	23.3	247	247, 291
Phyllanthusiin E (4)	19.0	291	247, 203
Corilagin (5)	19.5	633	453, 301, 633
Geraniin (6)	20.5	951	301, 633
Chebulagic acid (7)	21.2	953	301, 633
Phyllanthusiin C (8)	22.8	925	301, 633
Phyllanthusiin B (10)	24.2	969	301, 633
Phyllanthusiin U (11)	24.6	924	301, 633
Isostrictinin (2)	14.0	633	451, 301
Ellagic acid (12)	28.7	301	301
